# Effect of curcumin, betadine and chlorhexidine in gingival wound healing

**DOI:** 10.6026/973206300191153

**Published:** 2023-12-31

**Authors:** Ankita Agrawal, Ekka Rashmi Kiran, Singla Deepika, Patiksha Kumar, Deepak Kochar, Sidhu Ruhi, Bharat Gupta, Kapil Paiwal

**Affiliations:** 1 Department of Conservative and Endodontics, Buddha Institute of Dental Sciences and Hospital, Patna, Bihar, India; 2 Department of Oral Medicine and Radiology, Bhabha College of Dental Sciences, Bhopal, MP, India; 3 Department of Conservative Dentistry & Endodontics, Desh Bhagat Dental College & Hospital, Mandi Gobindgarh, Punjab, India; 4 Department of Oral Pathology & Microbiology, Government College of Dentistry, Indore, M.P., India; 5 Department of Periodontology, M.M. College of Dental Sciences & Research, Maharishi Markandeshwar (Deemed to be University), Mullana, Ambala, Haryana, India; 6 Oral Medicine and Radiology, MNDAV Dental College and Hospital, Tatul, Oachghat, Solan, Himachal Pradesh, India; 7 Department of Periodontics, MGM Dental College, Navi Mumbai, India; 8 Department of Oral & Maxillofacial Pathology, Daswani Dental College & Research Center, Kota, India

**Keywords:** Curcumin, chlorhexidine, human fibroblast viability, migration

## Abstract

The effect of chlorhexidine (CHX) digluconate, Betadine (BET), curcumin (CUR) on gingival wound healing is of interest to dental practitioners. Hence, we studied the average fibroblast viability % for each of the concentrations of CUR, BET and CHX over various time durations. It was found that mean percentage of viability of fibroblasts is high in CUR and low in CHX at all time periods while the mean percentage of viability of fibroblasts in BET 1% was greater than CHX but lower than CUR at all time periods. Thus, curcumin at a concentration of 0.003% demonstrates the least cytotoxicity for fibroblasts. Hence, it is the most effective bacterial suppression, and the best wound healing.

## Background:

Periodontal pathogens, their metabolites, and the host reaction interact intricately to cause the disease of periodontium, an on-going immune-inflammatory condition [1]. It is characterized by the lack of clinical adhesion and the breakdown of the ligament between the periodontal tissue and bone of the alveolar region. Innate immunity serves as the initial line of defense against microbial assault in the development of diseases of periodontium [2]. Toll-like receptors (TLRs), which are pathogen related pattern-recognizing receptors, have a particular response to the lipopolysaccharides constituent of gram-negative microorganisms [3]. The excessive stimulation of TLRs is thought to be the cause of the damage linked to periodontitis. Mediators of inflammation like nuclear factor (NF) κ-β and other inflammatory substances are activated by the LPS-stimulated TLR-4 [2- 3]. These mediators of inflammatory processes and reactive free radicals are responsible for the majority of periodontal damage. The basic objectives of treatment for periodontal disease are to stop bacterial/host-induced inflammation and keep the periodontium healthful. Plaque reduction is seen as the key to preventing this long-lasting condition, and nonsurgical as well as surgical approaches have been the mainstays of periodontal illness therapy. Along with mechanical removal of debris, various pharmacological plaque management treatments are utilised as adjuncts [1- 4]. Pharmaceutical plaque control treatments that work as an addition to manual treatment for periodontal disease include substances like triclosan, essential oils, and chlorhexidine (CHX) [4]. The pharmaceutical plaque prevention agent that is currently being studied and implemented the most is CHX [5]. The substantively of CHX is what gives it a better effect [6]. Although studies have shown that CHX is deadly to many different cell types, including fibroblasts of human skin, periodontal ligaments, and alveolar bone cells, it is nevertheless widely acknowledged as a safe and efficient antiplaque drug [7]. Inhibiting the processes of DNA synthesis and protein formation in fibroblasts as well as epithelial cells was possible at relatively low doses of CHX [8]. Research conducted in vitro has revealed that CHX has harmful effects on cell growth that are dose- along with time-dependent [9-10]. Although CHX is regarded as the pinnacle of effectiveness in pharmacologic plaque management, toxicity to fibroblasts is the main cause for worry.Hence, it is necessary to investigate an alternate agent that has equivalent antimicrobial and anti-inflammatory properties but lower fibroblast lethality.

Due to their safety, the use of herbal compounds as medicines has recently attracted significant attention in both medicine and dentistry across the globe. Medical study is increasingly placing more emphasis on turmeric, one of the most used home treatments. Curcumin, a hydrophobic polyphenol derived from the root system of curcumin longa, is the primary component of turmeric [11-13]. In addition to promoting healing of wounds, curcumin has immuno-modulatory, antioxidant, antibacterial and anti-inflammatory effects [14, 15,16,17]. Curcumin modulates NF-κβ when LPS activates TLR-4 in diseases of periodontium. Numerous cytokines are suppressed by curcumin, and several enzymes, including stimulated nitric oxide synthase and lipoxygenase, are down regulated. They have an extra benefit in treating periodontal disease since they are strong hunters of oxygen species that are reactive. Numerous periodontopathogens are inhibited by curcumin in a way that depends on the dose [18,19- 20].

A rinse with hydrogen peroxide that contains betadine (BET) (povidone iodine), a powerful antibacterial, can reduce the severity of gingivitis [21-22,23]. According to some research, when water is used as the irrigant for patients experiencing adult periodontitis, povidone iodine given by an ultrasonic device performs better in periodontal pockets with greater pocket depth than ultrasonic cleaning [23, 24,25]. Uncertainty surrounds the advantages of povidone iodine in the management of obstinate periodontitis. If used as a pre-procedural intra-sulcular irrigant, subgingival irrigation containing povidone iodine may lower the incidence of bacteremia; nevertheless, this approach is not advised for patients with elevated risk [26-27]. Therefore, it is of interest to document the effect of chlorhexidine (CHX) digluconate, betadine (BET) and curcumin (CUR) on gingival wound healing is of interest to dental practitioners.

## Materials and Methods:

The number of specimens in each category is 40. The estimated sample size for cytotoxic assessment was 480. The scratch wound assay used 160 samples. The estimated number of specimens for this in vitro investigation was 640.

## Experimental materials:

CHX digluconate 0.2 percent, betadine (BET) 0.5%& 1% and powdered curcumin (Sigma Aldrich, USA) were employed as the subject of the research's material of testing. The concentration of povidone iodine used was 0.5% and 1%. The concentrations of CHX applied in this research were ranging from 0.03% to 0.2%. The concentrations of CUR evaluated in this research were ranging from 0.003%, CUR 0.06% to 0.12% (Table 1). The following techniques were used to note the impact of CUR, CHX digluconate and BET upon human fibroblast cell longevity and emigration.

## Growing of fibroblasts:

The first source of human epidermal pulp embryonic stem cells was HiMedia, India Company (National Centre for Cell Sciences, India). They were grown in a mesenchymal embryonic stem cell multiplication medium called HiMesoXLTM. 10% foetal bovine serum that has been screened for mesenchymal stem cells, amphotericin B (2.5 g/ml), streptomycin (100 g/ml), sodium bicarbonate, L-glutamine and an antibiotic solutions comprising penicillin (100U/ml) are added as supplements. The cells had been trypsinized and grown in HiFibroXLTM fibroblast expansion media to transform stem cells into gingival fibroblast cells. Trypsinize consolidated monolayer cells that have been in place for two days before suspending them in growth-promoting media. 96-well tissue growth plans were seeded with a 100 µl cell mixture and cultivated at 37 degrees Celsius in a moistened five percent CO2 incubator. The cells produced using this method served as samples for the cytotoxicity assessment using MTT assay (3-(4,5-dimethylthiazol-2-yl)-2,5-diphenyltetrazolium bromide) and scratch wound assessment.

## MTT assay for evaluation of cytotoxicity:

Reassembled in 3-ml of phosphate buffer saline (PBS), a 15 mg MTT (Sigma, M-5655) is well dissolved before being filter sterilised. The fibroblasts were cultured for 24 hours with various doses of CUR, BET and CHX. All experimental as well as control wells received an addition of a 30-l regenerated MTT solution. The surface of the plate is subsequently shaken well before being left to incubate for 4 hours at 37 degrees Celsius in a moistened 5% CO2 chamber. The formazan crystals are solubilized by adding 100 µl of MTT solubilization fluid diethyl sulfoxide after the period of incubation has ended, and gently mixing the wells by piping upward and downward. At an emission wavelength of 540 nm, the absorption readings are calculated utilising a reader for micro plates. Forty samples were subjected to the same process, with similar preservation of all other parameters.

## Evaluation of scratch wounds:

A hygienic 1-ml pipette tip was used to make the incision wounds by passing it through the path that had been drawn on the fibroblast plates used for culture. The resulting cell monolayer was then three times flushed with PBS, the resultant detritus from 5 linear incisions was removed, and specimens with antibacterial concentrations of CHX (0.1%), BET 1% and CUR (0.003%) were added. These samples were incubated for 24 hours, 48 hours, and 72 hours. Additionally, the control plate underwent incubation without any test substances. Forty comparable plates of culture each for CHX, BET and curcumin were created. The percentage motility of fibroblast in the control, CHX, BET and CUR specimens at 24 hours, 48 hours, and 72 hours was used to quantify the wound healing.

## Statistical analysis:

For all the quantitative parameters, the mean (standard deviation) was computed. One-way ANOVA was used to calculate the average percentage of fibroblast proliferation and migration (wound healing) of control, CHX, BET, CUR at different points in time as well as the average percentage of fibroblast survival at the antibacterial levels of CHX, BET and curcumin. The average percentage of fibroblast survival at different levels of CUR, BET, CHX and control at every single time and the average percentage of fibroblast proliferation and migration at various time periods were compared within each category using a repeated-measures ANOVA. There was a Bonferroni post hoc test. The range of confidence was established at 95%, and the p value was established at 0.05.

## Results:

It was observed that the mean fibroblast viability decreased as the time duration increased for CHX at different concentrations as the time duration increased. However, the difference in observations was not meaningful statistically for each sample of CHX. It was also observed that the mean fibroblast viability decreased as the concentration of CHX increased (Table 2). It was observed that mean percentage of viability of fibroblasts decreased as the time duration increased for all CUR at all different concentrations. The values were meaningful statistically in case of CUR 0.003%, CUR 0.03%, and CUR 0.1%, 0.12%. It was also observed that the mean percentage of viability of fibroblast decreased as the concentration of CUR increased (Table 3).

It was observed that the mean fibroblast viability decreased as the time duration increased for BET at different concentrations as the time duration increased. However, the difference in observations was not meaningful statistically for each sample of BET. It was also observed that the mean fibroblast viability decreased as the concentration of BET increased (Table 4).

The values of mean percentage of viability of CHX 0.1% (the antibacterial concentration of Chlorhexidine), CUR 0.003% (the antibacterial concentration of curcumin) and BET 1% (the antibacterial concentration of BET 1%) are being shown in Table 3. It was found that mean percentage of viability of fibroblasts was maximum in CUR and minimum in CHX at all time periods. The mean percentage of viability of fibroblasts in BET 1% was greater than CHX but lower than CUR at all time periods The findings were significant statistically.(p=0.0001)(Table 5)

It was observed that proliferation and migration of fibroblasts (wound healing) was greatest in curcumin specimens at 24 hours, 48 hours and 72 hours duration and minimum in CHX. The proliferation and migration of fibroblasts (wound healing) in BET was greater than CHX but lower than CUR.The findings were meaningful statistically. The values of proliferation and migration of fibroblasts (wound healing) increased in all specimens on increasing time duration significantly (Table 6).

## Discussion:

The antiplaque effects of CHX and CUR mouth rinse were evaluated elsewhere [17- 19]. They showed that CUR mouth rinse can be used as an alternate antiplaque agent to CHX. However, there was no attempt to standardize curcumin concentrations in terms of MIC. Despite being a naturally produced polyphenol, CUR is cytotoxic and causes cell death at high concentrations, a feature that has led to its usage as an anticancer medication. As shown elsewhere [20], unlike to CHX, curcumin's antimicrobial concentration (0.003%) demonstrates less fibroblast cytotoxicity and superior wound healing properties. Curcumin is a promising chemical plaque management agent that is less cytotoxic, economical, safe, and accessible, as well as having potential advantages in wound healing.

Stopping bacterial/host-induced inflammation and maintaining the periodontium's health are the main goals of treatment for periodontal disease. The bases of periodontal disease therapy have been nonsurgical as well as surgical therapies, with plaque reduction being viewed as the key to treating this chronic condition [21- 22]. Several pharmaceutical plaque management treatments are used as adjuncts to mechanical debris removal. Pharmaceutical plaque control treatments for periodontal disease include ingredients including triclosan, essential oils, and chlorhexidine (CHX), which are effective in addition to manual treatment. CHX is now the pharmacological plaque prevention medication that is studied and used the most. CHX works better because of its applicability [23- 24]. Turmeric's main ingredient is curcumin, a hydrophobic polyphenol obtained from curcumin longa's root system. Curcumin contains immuno-modulatory, antioxidant, antimicrobial, and anti-inflammatory properties in addition to aiding in wound healing [25,26]. When LPS stimulates TLR-4 in periodontal disorders, curcumin regulates NF-κβ [14]. Curcumin inhibits many cytokines and down regulates a number of enzymes, including lipoxygenase and stimulated nitric oxide synthase [27-28]. Since they are effective hunters of reactive oxygen species, they have an added advantage in the treatment of periodontal disease. Curcumin inhibits a variety of periodontopathogens in a dose-dependent manner [29].

Povidone iodine is a secure antiseptic that doesn't seem to slow down wound healing or create bacteria that are resistant to it. Data indicates that using povidone in the treatment of some periodontal disorders may be advantageous. However, this evaluation is based on a small sample size. Hence, more clinical trials are required to confirm it.

Chlorhexidine has been found to inhibit human fibroblast binding to root surfaces and to impact cellular growth [6], as well as total protein synthesis in vitro in recent studies utilising cells from the periodontium [7]. Additionally it [8] is hypothesized that chlorhexidine will cause a dose-related decrease in proliferation of cells and that human gingival fibroblasts' in vitro output of both collagen as well as non-collagen proteins can be significantly reduced at chlorhexidine concentrations that have a small impact on the proliferation of cells. In a recent study [9], it was established that chlorhexidine is detrimental to human gingival fibroblasts in vitro. It also showed that fibroblast cells are cytotoxic to povidone-iodine at 0.5% and 1% doses. This outcome was consistent with a prior investigation [14], which showed that canine embryonic fibroblasts were killed in vitro by povidone-iodine at concentrations of 5.0% to 0.05%. The results found similarity with previous data [15, 16], which showed the cytotoxic action of betadine on human gingival fibroblasts.

## Conclusion:

The effect of chlorhexidine (CHX) digluconate, Betadine (BET), curcumin (CUR) on gingival wound healing is of interest to dental practitioners. Hence, a comparative study was completed. Data shows that curcumin at a concentration of 0.003% shows best wound healing and least cytotoxicity as compared to betadine and chlorhexidine as shown elsewhere [20]. Data also shows that it has significant antibacterial properties. Curcumin is less cytotoxic and it is a conveniently accessible antiplaque agent.

## Figures and Tables

**Table 1:** The distribution of specimens

**Mouthrinse**	**Concentration**	**Number(n)**
BET	0.50%	40
BET	1%	40
CHX	0.03%	40
CHX	0.06%	40
CHX	0.10%	40
CUR	0.00%	40
CUR	0.06%	40
CUR	0.10%	40
CUR	0.12%	40

**Table 2:** The mean values of viability of fibroblasts in Chlorhexidine specimens at different concentrations and different time durations

		**0.03%**	**0.06%**	**0.1%**	**0.12%**	**0.2%**
Mean percentage of fibroblast Viability (%)	1 minute	78.11	59.61	49.76	43.86	37.24
	2 minute	72.289	58.65	48.55	41.02	34.94
	4 minute	66.75	53.16	41.56	38.37	32.81
	6 minute	64.34	50.03	40.84	36.09	32.32
	8 minute	58.23	44.95	40.35	35.5	31.25
	10 minute	54.73	44.44	39.86	35.33	31.08
Intragroup comparison	F	7.543	1.746	5.547	2.78	2.978
	df	1.678	3.061	1.594	1.286	1.693
	P value	0.075	0.271	0.214	0.217	0.296

**Table 3:** The mean values of viability of fibroblasts in Curcumin at different concentrations and different time durations

		**0.003%**	**0.03%**	**0.06%**	**0.1%**	**0.12%**
Mean percentage of Fibroblast Viability (%)	1 minute	99.05	90.78	72.2	56.44	39.34
	2 minute	90.81	85.69	67.7	52.61	36.88
	4 minute	77.042	75.74	64.96	50.97	35.79
	6 minute	74.05	72.89	63.96	49.85	34.31
	8 minute	72.42	70.495	63.73	46.77	34.21
	10 minute	68.67	65.45	63.57	45.97	32.93
Intragroup comparison	F	97.23	56.21	4.124	11.71	7.549
	df	1.471	1.223	1.567	1.971	1.256
	P value	0.001*	0.014*	0.146	0.036*	0.039*

**Table 4:** The mean values of viability of fibroblasts in Betadine (BET) specimens at different concentrations and different time durations

		**0.5%**	**1%**
Mean percentage of fibroblast Viability (%)	1 minute	88.12	69.67
	2 minute	82.29	68.76
	4 minute	76.72	63.16
	6 minute	74.36	60.14
	8 minute	68.35	54.06
	10 minute	65.84	54.47
Intragroup comparison	F	8.654	2.857
	df	2.729	4.172
	P value	0.087	0.382

**Table 5:** The values of viability of fibroblasts of chlorhexidine 0.1%, curcumin 0.003% and BET 1% at different time duration

		**CHX 0.1%**	**CUR 0.003%**	**BET 1%**
Mean percentage of Fibroblast viability (%)	1 minute	49.76	99.05	69.67
	2 minute	48.55	90.81	68.76
	4 minute	41.56	77.042	63.16
	6 minute	40.84	74.05	60.14
	8 minute	40.35	72.42	54.06
	10 minute	39.86	68.67	54.47
Intergroup comparison	F		102.841	
	df		11.24	
	P value		0.0001*	

**Table 6:** The values of proliferation and migration of fibroblasts (healing of wound) at different time duration

		**Control**	**CHX 0.1%**	**CUR 0.003%**	**BET 1%**
Mean percentage of proliferation and migration of fibroblasts (healing of wound)	24 hr	2.82	8.94	17.7	12.4
	48 hr	14.43	9.67	26.59	17.56
	72 hour	22.14	10.45	40.93	24.76
Intragroup comparison	F	4.75E+11	1.57E+11	3.17E+12	2.68E+11
	df	1.328	2.551	2.28	2.432
	P value	0.000*			
Intergroup comparison	F		102.841		
	df		11.24		
	P value		0.0001*		
